# Stable Core–Shell ZIF-8@TPPa Hybrids: Synthesis and Enhanced Herbicide Removal from Water

**DOI:** 10.3390/molecules31111799

**Published:** 2026-05-24

**Authors:** Zeyuan Li, Zhenzhen Liu, Xiangping Lin, Mengyuan Ge, Nannan Wu, Xinquan Wang, Yuteng Zhou, Shuchun Wu, Wei Ding, Peipei Qi

**Affiliations:** 1 College of Plant Protection, Northeast Agricultural University, Harbin 150030, China; zeyuan1214@gmail.com (Z.L.); linxiangping2018@163.com (X.L.); gemengyuan0413@163.com (M.G.); 2 State Key Laboratory for the Quality and Safety of Agro-Products & Institute of Agro-Product Safety and Nutrition, Zhejiang Academy of Agricultural Sciences, Hangzhou 310021, China; 18768479497@163.com (Z.L.); wunannan4089@163.com (N.W.); wangxq@zaas.ac.cn (X.W.); zhouyuteng1999@163.com (Y.Z.); 3 Key Laboratory of Biomarkers and In Vitro Diagnosis Translation of Zhejiang Province, School of Laboratory Medicine and Bioengineering, Hangzhou Medical College, Hangzhou 310053, China; w_sc@163.com

**Keywords:** ZIF-8@TPPa, herbicides, adsorption, aqueous environment

## Abstract

The excessive use of herbicides in agricultural fields has emerged as a critical environmental concern. This study innovatively synthesized a ZIF-8@TPPa composite through a solvothermal method for the efficient removal of herbicides from aqueous environment. The material exhibited remarkable adsorption capacities for butachlor (232.56 mg/g), anilofos (188.68 mg/g), and pendimethalin (285.71 mg/g), along with excellent acid–base stability (pH 3–9), strong anti-ion interference capability, and good reusability (adsorption efficiency >80% after five cycles). The adsorption processes were well-described by the two isotherm models and the pseudo-second-order model, indicating that the dominant mechanism is a synergistic effect between monolayer chemical adsorption and multilayer physical adsorption, primarily driven by π-π stacking, hydrogen bonding, and coordination. The material maintained outstanding adsorption efficiency (>85%) in real water samples (tap water, seawater, and river water). This study not only provides a sustainable and effective strategy for herbicide remediation from aqueous environment but also expands the practical applications of MOF@COF in aqueous environment.

## 1. Introduction

Herbicides, particularly amide, aniline, and organophosphorus types, are the primary agents used for weed control to boost crop yields and have significantly expanded the global market [1,2,3]. However, their environmental risks have become a prominent issue due to their slow biodegradation and accumulation in surface water and groundwater systems [4]. These herbicides migrate via surface runoff, soil infiltration, and atmospheric deposition, leading to contamination in rivers, with butachlor reported at concentrations of up to 400 μg/L [5]. Ultimately, these herbicide residues disrupt ecosystems through bioaccumulation and pose serious health threats, including neurotoxicity, respiratory damage, and cancer [6,7,8]. Therefore, developing reliable methods for removing herbicide residues from aquatic systems is critical.

Adsorption is an effective method for removing herbicides from the environment owing to its simplicity, low cost, straightforward regeneration, and high automation potential [9,10,11,12]. However, existing adsorbents still exhibit certain limitations in adsorption capacity. For instance, Ch/PVA-Ag nanocomposites and Fe_3_O_4_-MWCNT-ZIF-67, specifically designed for butachlor adsorption, exhibit a maximum adsorption capacity of 8.24–23.8 mg/g [13]. In addition, conventional adsorbents suffer from limited active sites and uniform pore structures, making it difficult to effectively adsorb herbicides with complex molecular structures. Therefore, there is an urgent need to develop highly efficient adsorbents for herbicides removal.

Metal–organic frameworks (MOFs) and covalent organic frameworks (COFs) are two emerging classes of adsorbents that have garnered considerable attention on account of their large specific surface areas, adjustable pore sizes, and strong adsorption capabilities [14,15]. Notably, these two materials exhibit distinct pore structure characteristics. MOFs primarily feature micropores, which offer abundant active sites but poor water stability. COFs typically possess mesopores, conferring enhanced pore stability in aqueous environments yet limiting adsorption capacity for macromolecular substances [16,17]. Recent studies have focused on fabricating MOF@COF composites to address these pore structure discrepancies. Luo [18] developed a novel MOF@COF (UiO-67@MCA) that achieved a 1.25-fold higher adsorption for 2,4-dichlorophenoxyacetic acid and glyphosate than UiO-67. While MOF@COF materials enhance herbicide adsorption, current research remains largely confined to single herbicides or a single class of herbicides, limiting their practical applicability. For instance, UiO-66@COF-V [19] only enriches phenoxycarboxylic acid herbicides. Therefore, a broad-spectrum MOF@COF adsorbent capable of simultaneously removing amide-, aniline-, and organophosphorus-based herbicides is critically needed.

ZIF-8, a well-established MOF, exhibits high adsorption capacity for pollutants owing to its excellent specific surface area and uniform microporous structure [20,21]. However, under acidic conditions (pH 2–6), its imidazole ligands undergo protonation, leading to framework degradation and a sharp decline in adsorption capacity [22]. Hence, coating ZIF-8 with a stable COF can resolve its insufficient stability, while the hierarchical pore structure is likely to contribute to an increase in the adsorption rate. Based on the properties of the three herbicides, p-phenylenediamine (Pa) and 2,4,6-trihydroxybenzene-1,3,5-tricarbaldehyde (TP) were selected to prepare TPPa COF as a protective layer on the surface of ZIF-8. TPPa has a conjugated aromatic system and electron-rich nitrogen sites, which may improve adsorption capacity through hydrogen bonding and π-π stacking [23]. Therefore, the ZIF-8@TPPa composite, which combines ZIF-8 and TPPa, not only possesses enhanced structural stability and more abundant active sites but also exhibits a hierarchical pore structure that facilitates rapid diffusion and mass transfer of herbicide molecules, leading to significantly improved adsorption performance.

In this study, butachlor (amide-based), anilofos (organophosphorus-based), and pendimethalin (aniline-based) (Table S1) were selected to assess the adsorption capability of ZIF-8@TPPa. The adsorption efficiency was systematically investigated under varying contact times, adsorption dosages, and initial pH conditions. The adsorption behavior was analyzed using typical adsorption kinetic, isotherm, and thermodynamic models. Furthermore, the adsorption mechanisms were elucidated using X-ray photoelectron spectroscopy (XPS) and Fourier-transform infrared spectroscopy (FT-IR). Additionally, both reusability and ion interference assessments were conducted to validate its applicability.

## 2. Results and Discussion

### 2.1. Characterization of ZIF-8@TPPa

**Figure 1:**
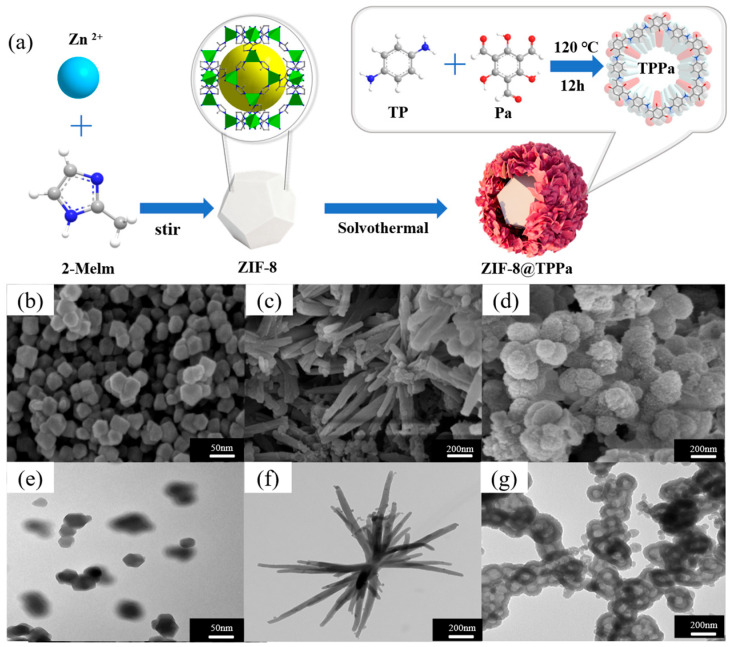
Schematic illustration of ZIF-8@TPPa synthesis (**a**); TEM and SEM images of ZIF-8 (**b**,**e**), TPPa (**c**,**f**), and ZIF-8@TPPa (**d**,**g**), respectively.

The morphological features of ZIF-8, TPPa, and ZIF-8@TPPa were systematically characterized by SEM and TEM. As shown in Figure 1b,e, the as-prepared ZIF-8 exhibited a regular dodecahedral structure with smooth surfaces and a uniform particle size distribution, which is consistent with previous studies [24]. Figure 1c,f reveal that TPPa displayed a rod-shaped morphology and self-assembled into divergent bouquet-like clusters. As demonstrated in Figure 1d,g, ZIF-8@TPPa exhibited a well-defined core–shell morphology. The inner ZIF-8 core appears as a dark and compact particle, while the outer TPPa shell forms a continuous light layer wrapped around the core, indicating the successful formation of the core–shell structure [25,26,27]. Moreover, the energy-dispersive spectroscopy (EDS) elemental mapping images reveal a uniform distribution of C, N, and O across the entire particle, while the Zn signal is localized at the center. This distinct elemental distribution verifies the core–shell architecture, where a ZIF-8 core is encapsulated by a TPPa shell (Figure S1).

**Figure 2:**
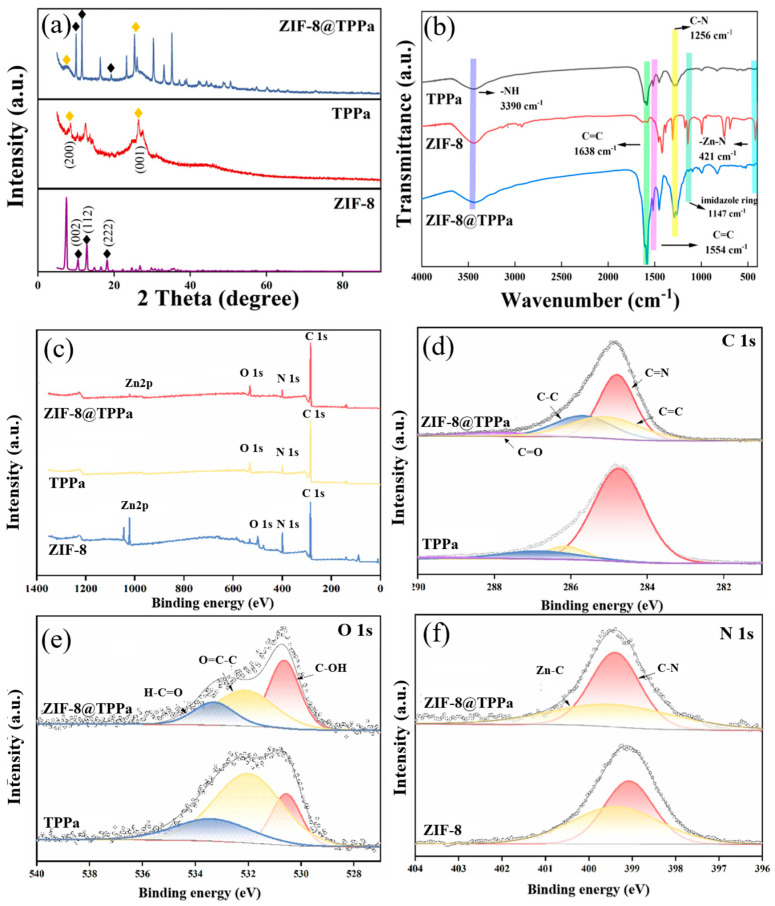
XRD patterns of ZIF-8, TPPa, and ZIF-8@TPPa (black rhombus: characteristic peaks of ZIF-8; yellow rhombus: characteristic peaks of TPPa) (**a**); FT-IR spectra of ZIF-8, TPPa, and ZIF-8@TPPa (**b**); XPS survey spectra of ZIF-8, TPPa, and ZIF-8@TPPa (**c**); fine spectra of C 1s (**d**), O 1s (**e**), and N 1s (**f**).

XRD measurements were performed to characterize the crystalline structure of ZIF-8, TPPa, and ZIF-8@TPPa. As depicted in Figure 2a, the XRD pattern of ZIF-8 exhibited characteristic peaks at 2θ = 10.4°, 13.0°, and 17.4°, assigned to the (002), (112), and (222) crystal planes, respectively, confirming its high crystallinity [28]. Meanwhile, the TPPa component showed distinct peaks at 2θ = 8.3°and 26.7°, assigned to the (200) and (001) crystal planes [29]. Notably, ZIF-8@TPPa exhibited a slight rightward shift in the (222) crystal plane diffraction peak. This phenomenon is commonly attributed to the interaction between ZIF-8 and the introduced mixed organic ligands [30]. Furthermore, the XRD pattern of ZIF-8@TPPa clearly retained all characteristic peaks of both the ZIF-8 and TPPa components, demonstrating the successful formation of the composite.

The functional groups of the three materials were characterized via FT-IR. As illustrated in Figure 2b, the FT-IR spectrum of the composite showed characteristic peaks at 3300–3500 cm^−1^ and 1638 cm^−1^, which correspond to a weak -NH stretching vibration peak and a typical -C=C absorption peak, respectively, confirming the presence of TPPa [31]. Additionally, the peaks at 1554 cm^−1^, 1256 cm^−1^, 1147 cm^−1^ and 421 cm^−1^ were ascribed to the -C=C, -C-N, imidazole ring and Zn-N vibrations of the imidazole ring in ZIF-8 [32]. The FT-IR results revealed that the ZIF-8@TPPa composite retained both the aromatic rings of TPPa and the imidazole ring coordination structure of ZIF-8, with no shifts in peak positions or losses in peak intensities observed. This indicates that no destruction of critical structures occurred during the synthesis process, thereby laying a solid foundation for the high stability and excellent adsorption capacity of the resulting material. Hence, the results verified the successful functionalization of ZIF-8 with TPPa.

The surface compositions and chemical states of the three materials were carried out by XPS analysis. Figure 2c presents the XPS spectra of the surface elemental valence states of the three materials, among which the spectrum of the ZIF-8@TPPa composite evidences the coexistence of characteristic elements derived from both ZIF-8 and TPPa. The high-resolution C 1s and O 1s spectra of TPPa and ZIF-8@TPPa are shown in Figure 2d and Figure 2e, respectively. In the high-resolution C 1s spectrum, four distinct peaks were observed at binding energies of 284.55 eV (C=C), 284.21 eV (C=N), 285.20 eV (C-C), and 287.82 eV (C=O) [33]. The O 1s spectrum exhibited three peaks at 530.64 eV (C-OH), 532.12 eV (O=C-C), and 533.30 eV (O=CH). These results were in accordance with previous studies [34]. In the N 1s spectra of ZIF-8 and ZIF-8@TPPa (Figure 2f), the peaks at 399.38 eV and 399.68 eV were assigned to C-N and Zn-N bonds [35], respectively, further verifying the successful formation of ZIF-8. Overall, all elements in the composite could be clearly traced to the two precursors (ZIF-8 and TPPa). Furthermore, the highly matched XPS binding energies between the two precursors and the composite demonstrated that the active sites and core structures of both precursors were well preserved during the synthesis process. This result, which is consistent with the FT-IR characterization results, further confirms the successful synthesis of the ZIF-8@TPPa composite.

The specific surface area, pore size distribution, and adsorption–desorption behaviors of ZIF-8, TPPa, and ZIF-8@TPPa were analyzed via Brunauer–Emmett–Teller (BET) analysis. As exhibited in Figure S2, the N_2_ adsorption–desorption isotherm of ZIF-8 exhibited a Type I adsorption isotherm, which is characteristic of microporous materials [36]. In contrast, TPPa displayed a Type IV isotherm with an H3 hysteresis loop, confirming its mesoporous structure [37]. As shown in Table S2, the specific surface area of ZIF-8@TPPa (162.2 m^2^/g) was significantly lower than that of pristine ZIF-8 (1700.8 m^2^/g). This substantial reduction is primarily attributed to the formation of a dense TPPa shell on the ZIF-8 surface, which results in the partial blockage of the microporous channels in the ZIF-8 core [38]. However, as shown in Figure S2c, the N_2_ adsorption–desorption isotherm of ZIF-8@TPPa displays distinct adsorption patterns across different relative intervals. In the low-pressure region (*P*/*P*_0_ < 0.1), the adsorption capacity undergoes a rapid increase followed by a plateau, which is characteristic of the rapid filling of microporous structures. In the medium-to-high pressure range (*P*/*P*_0_ = 0.4–0.9), a further increase in adsorption capacity is observed, accompanied by a well-defined closed hysteresis loop, a phenomenon attributed to capillary condensation within mesopores originating from TPPa. This observation precisely confirms the successful coating of TPPa onto ZIF-8 rather than the absence of the ZIF-8 core. Consequently, the N_2_ adsorption–desorption isotherm of ZIF-8@TPPa integrates the features of both Type I and Type IV isotherms, thus providing compelling evidence for the successful synthesis of ZIF-8@TPPa.

Notably, despite the substantial reduction in BET surface area, ZIF-8@TPPa exhibits significantly higher adsorption removal rates than pristine ZIF-8 and TPPa for all three tested herbicides (Table S1). ZIF-8@TPPa achieves removal rates of 99%, 93%, and 90% for pendimethalin, anilofos, and butachlor, respectively. This superior performance arises from the hierarchical micro-mesoporous structure, where the remaining micropores and the newly generated mesopores offer efficient diffusion channels for herbicides and optimize pore size matching. This hierarchical structure favors mass transfer and molecular accessibility. Meanwhile, the TPPa coating introduces abundant highly active adsorption sites, including aromatic rings, hydroxyl groups, amino groups, and conjugated skeletons. These sites establish strong multi-point interactions (*π-π* stacking, hydrogen bonding, and hydrophobic effects) with herbicide molecules. A surface with fewer but stronger and more selective sites can outperform a high-area surface with weak interactions. Moreover, the core–shell ZIF-8@TPPa integrates complementary functions that address the individual limitations of the pristine materials, including weak metal coordination, poor stability in aqueous environments, and limited dispersibility, further enhancing its overall adsorption performance.

### 2.2. Adsorption Performance

In this research, the solution pH, adsorbent dose, and contact time were systematically investigated. The optimization trials were all carried out in 20 mL aqueous solutions, with the initial herbicides concentration maintained at 10 mg/L. The adsorption efficiencies of ZIF-8@TPPa toward the three herbicides were calculated and used as the main indicators for assessing the adsorption process.

#### 2.2.1. Effects of pH on Adsorption

**Figure 3:**
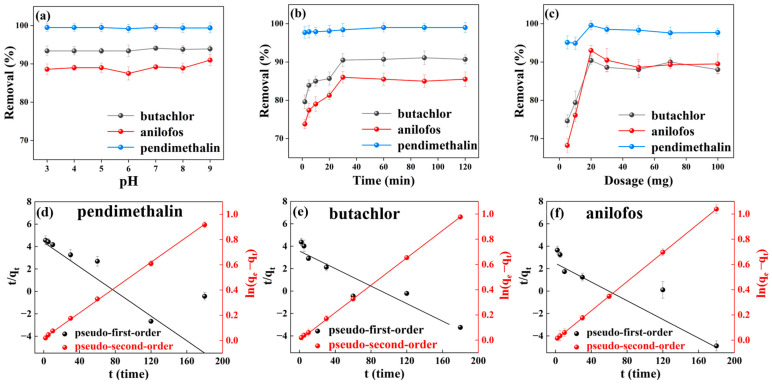
Effects of pH values (**a**), contact time (**b**) and dosage (**c**) on the adsorption efficiency of three herbicides (adsorption conditions: T = 298 K, rotating speed = 200 rpm, initial concentration of herbicides C_0_ = 10 mg/L); kinetic model fitting of pendimethalin (**d**), butachlor (**e**), and anilofos (**f**) by pseudo-first-order and pseudo-second-order models.

The pH of aqueous systems can influence adsorption by modulating the surface charges of both target pollutants and adsorbents. As exhibited in Figure 3a, the adsorption efficiency of butachlor, anilofos, and pendimethalin ranged from 87.5% to 91.0%, 93.4% to 94.1%, and 99.4% to 99.5%, respectively, demonstrating that the adsorption performance of ZIF-8@TPPa for herbicides remained stable within the pH range of 3–9. This finding confirms that the structure of ZIF-8@TPPa did not break down in acid–base environments, suggesting that ZIF-8@TPPa has excellent acid–base stability. Thus, pH 7 was selected for subsequent experiments as it is representative of natural water conditions.

#### 2.2.2. Effects of Adsorbent Dosage

Adsorbent dosage is a critical parameter affecting the contact efficiency between adsorbent surfaces and herbicides. The influence of ZIF-8@TPPa dosages (5–100 mg/20 mL) was examined. As shown in Figure 3b, the adsorption efficiency of the three herbicides increased gradually within the adsorbent dosage range of 5–20 mg, reaching 90.4%, 93.0%, and 99.6% from initial values of 68.2%, 74.6%, and 95.1%, respectively. This may be due to the greater availability of active adsorption sites. However, the adsorption efficiency remained nearly unchanged when the dosage exceeded 20 mg (20–100 mg), demonstrating that the adsorption process achieved equilibrium. Hence, 20 mg was selected as the optimum dosage.

#### 2.2.3. Effects of Contact Time

The influence of contact time on the adsorption efficiency of herbicides was studied. As presented in Figure 3c, when the contact time ranged from 2 to 30 min, the adsorption efficiencies of butachlor, anilofos, and pendimethalin increased from 73.8% to 86.0%, 79.6% to 90.5%, and 97.7% to 98.4%, respectively. When the contact time reached 30 min, the change in adsorption efficiency for herbicides did not exceed 3%, indicating that adsorption equilibrium was essentially attained at 30 min. Therefore, a contact time of 30 min was selected for subsequent tests.

### 2.3. Adsorption Kinetics and Isotherms

To elucidate the adsorption kinetics of ZIF-8@TPPa toward herbicides, the experimental data were analyzed using both PFO and PSO kinetic models. As summarized in Table 1 and Figure 3e,f, the adsorption data correlated poorly with the PFO model (R^2^ = 0.8928, 0.8578, and 0.7719) but exhibited an excellent fit to the PSO model (R^2^ = 0.9999, 0.9999, and 0.9995), indicating that chemisorption act as the dominant mechanism [39]. Additionally, the experimental adsorption capacities of ZIF-8@TPPa for the herbicides were 184.4, 173.2, and 197.0 mg/g, respectively, which were in good proximity to the calculated adsorption capacities in Table 1 (185.2, 172.4, and 201.1 mg/g). The excellent agreement between the experimental and theoretical data verified the reliability of the PSO kinetic model for characterizing the adsorption behaviors.

**Figure 4:**
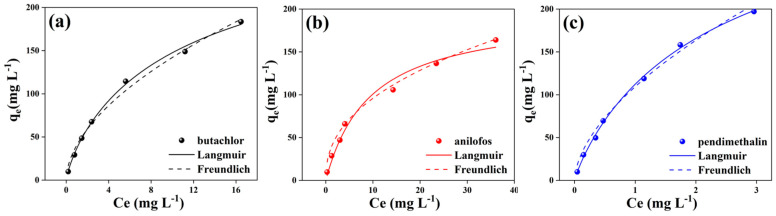
Adsorption isotherm models for butahclor (**a**), anilofos (**b**), and pendimethalin (**c**).

Isotherm studies were conducted to elucidate the adsorption behavior. Herein, the experimental data were fitted with both the Langmuir and Freundlich models. The Langmuir model is conventionally utilized to describe monolayer adsorption, whereas the Freundlich model characterizes multilayer adsorption on heterogeneous surfaces [40]. As summarized in Figure 4a–c and Table S3, the Freundlich model exhibited marginally better fitting performance (R^2^ = 0.9866, 0.9825, and 0.9905) than the Langmuir model (R^2^ = 0.9863, 0.9767, and 0.9785). The high similarity in R^2^ values suggests that the adsorption process may involve both monolayer chemisorption and multilayer physisorption, rather than being dominated by a single mechanism [41]. Additionally, the 1/n values ranged from 0.5714 to 0.7143, indicating that the adsorption was heterogeneous and favorable [42].

### 2.4. Adsorption Thermodynamics

The thermodynamic behavior of the adsorption procedure was examined through temperature-controlled experiments. Key parameters, including ΔH, ΔS, and ΔG, were derived to assess the effects of temperature and spontaneity of adsorption. As shown in Figure S3, the van’t Hoff plot (lnK_0_ against 1/T) exhibited an excellent linear correlation, and the relevant thermodynamic parameters were calculated and compiled in Table S4. The negative values of ΔG, ranging from −12.8 to −5.03 kJ/mol, confirmed the thermodynamically spontaneity of the adsorption. Positive ΔH values (21.25–56.4 kJ/mol) suggested an endothermic adsorption mechanism, consistent with chemisorption behavior [43]. In addition, as the adsorption temperature increased, the ΔG values decreased, demonstrating that the procedure is more favorable at higher temperatures. The positive ΔS values suggested increased entropy at the solid–liquid interface, which could result from the release of hydrated water molecules or structural reorganization of the ZIF-8@TPPa surface during adsorption [44]. These results demonstrated that the adsorption procedure is endothermic, spontaneous, and entropically driven, with higher temperatures enhancing adsorption.

### 2.5. Adsorption Mechanism

**Figure 5:**
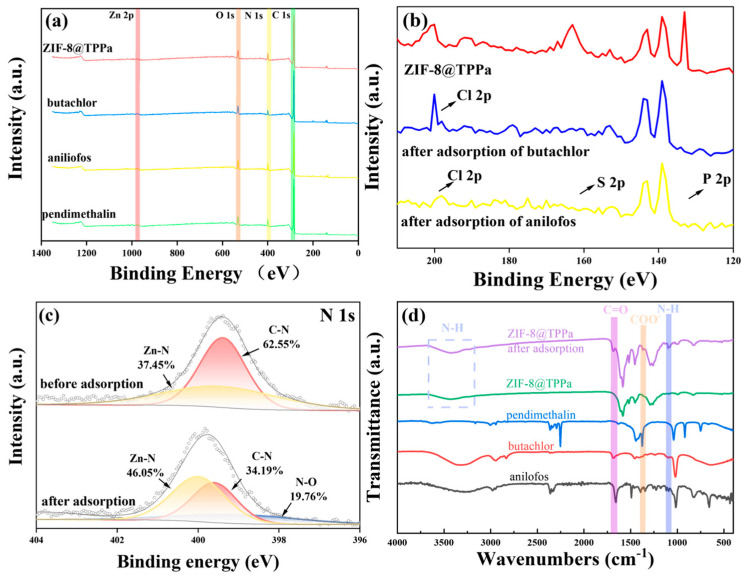
XPS survey spectra (**a**–**c**) and FT-IR spectra (**d**) of ZIF-8@TPPa before and after butachlor, anilofos, and pendimethalin adsorption.

The adsorption mechanism of ZIF-8@TPPa was systematically elucidated through FT-IR and XPS. Notably, pendimethalin shares a similar elemental composition with ZIF-8@TPPa, requiring high-resolution XPS to confirm its adsorption. As shown in Figure 5a, after the adsorption of butachlor and anilofos, the new binding energy peaks of Cl 2p, S 2p, and P 2p appeared in the XPS survey spectra of ZIF-8@TPPa, which confirmed the successful adsorption of butachlor (Cl-containing group) and anilofos (S- and P-containing groups). As presented in Figure 5b, after the adsorption of pendimethalin, a new peak at 400.00 eV (N-O group) appeared in the N 1s spectrum. Moreover, the peaks associated with C-N (399.39 eV) and Zn-N (399.42 eV) shifted to 398.96 eV and 399.60 eV, respectively. These shifts may suggest coordination interactions between the open Zn sites of ZIF-8@TPPa and N-containing functional groups in the herbicides. It is plausible that the Zn sites were occupied by Lewis basic N groups during adsorption, thereby facilitating the herbicide adsorption process [45].

The normalized Fourier-transform infrared (FT-IR) spectra of ZIF-8@TPPa before and after herbicide adsorption may provide additional mechanistic insights (Figure 5c). The enhanced peak at 1367 cm^−1^, tentatively assigned to the -COO^−^ group of the herbicides, suggests that herbicide adsorption had occurred. In addition, the new peak appearing at 1685 cm^−1^ corresponds to C=O stretching, which originates from the successful adsorption of butachlor or anilofos. The aromatic rings of these herbicides can form π-π interactions with the conjugated framework of ZIF-8@TPPa [46]. Moreover, the peak around 1100 cm^−1^ exhibited an obvious change after adsorption. The N-H stretching band of ZIF-8@TPPa shifted from 3430 cm^−1^ to 3428 cm^−1^, and the full-width at half-maximum (FWHM) broadened from approximately 120 cm^−1^ to 150 cm^−1^. These changes are commonly considered to be indicative of hydrogen bond formation involving N-H groups [47].

The adsorption experiments revealed an adsorption efficiency trend among the three herbicides: pendimethalin > butachlor > anilofos, which may be attributed to their distinct molecular architectures and chemical properties. Pendimethalin contains –NO_2_ and –NH– groups that facilitate hydrogen bond formation and also features a compact monoaromatic conjugated system. Its low steric hindrance and minimal diffusion resistance enable rapid migration to active sites, predominantly through π-π stacking, thus exhibiting the highest adsorption efficiency [48]. In contrast, butachlor can form weak hydrogen bonds with the adsorbent surface via its C=O group. However, it contains a longer n-butoxy chain, resulting in much greater steric hindrance than pendimethalin, which slows its penetration into the microporous channels of ZIF-8 and thereby reduces adsorption kinetics [49]. Anilofos possesses P=O groups that may act as Lewis bases for coordination with the open Zn sites of ZIF-8. Yet, the bulky phosphate group together with the longer propylthio chain leads to a large molecular structure with the highest steric hindrance. This not only prevents its entry into ZIF-8 but may also cause local accumulation on the TPPa shell, further reducing the effective binding between herbicide molecules and active sites.

### 2.6. Impact of Interfering Ions, Actual Water Application, and Reusability

The presence of coexisting ions in aqueous systems may significantly affect adsorption processes. To evaluate the environmental applicability of ZIF-8@TPPa, the effects of various ions (NaCl, Na_2_SO_4_, K_2_CO_3_, and H_3_PO_4_) at different concentrations (0–4%) on the adsorption efficiency of butachlor, anilofos, and pendimethalin were investigated. As presented in Figure S4, ZIF-8@TPPa exhibited excellent anti-interference capability, maintaining adsorption efficiencies of over 82% under the tested conditions. Notably, compared with neutral salts (NaCl and Na_2_SO_4_) and the weakly alkaline salt (K_2_CO_3_), anilofos adsorption was slightly more affected in the presence of acidic H_3_PO_4_ ions. This is likely due to their similar structural motifs, which lead to competitive adsorption. Nevertheless, the overall adsorption efficiency still remained above 80%. These results demonstrated that ZIF-8@TPPa can effectively adsorb herbicides in complex aqueous environments.

Furthermore, the practical performance of ZIF-8@TPPa was assessed by evaluating its adsorption efficiency toward herbicides in river water, seawater, and tap water. As shown in Figure S5, ZIF-8@TPPa exhibited high adsorption efficiency for butachlor (>85%), anilofos (>88%), and pendimethalin (>96%) across all tested water types. These results showed the practical applications of ZIF-8@TPPa in real aqueous environments.

Reusability was examined over five adsorption–desorption cycles. As shown in Figure S6, following five adsorption–desorption cycles, the adsorption capacities for all three herbicides still remained over 80%. Notably, the adsorption efficiency of pendimethalin remained as high as 97%. Furthermore, structural stability after regeneration was confirmed by XRD and SEM analyses (Figure S7). The SEM image confirmed that the post-desorption material retained a well-defined core–shell morphology without apparent degradation. Correspondingly, the XRD pattern showed no significant shift, broadening, or loss in intensity of the characteristic diffraction peaks, indicating that the crystalline structures of both the ZIF-8 and TPPa components remained intact. These results demonstrate the robust stability and excellent reusability of ZIF-8@TPPa.

## 3. Materials and Methods

### 3.1. Materials

N, N-dimethylformamide (DMF, >99%) were purchased from Sinopharm Chemical Reagent Co., Ltd. (Shanghai, China). Acetonitrile and methanol of High-Performance Liquid Chromatography (HPLC) grade were provided by Merck (Darmstadt, Germany). The herbicides butachlor (99%), anilofos (97%), and pendimethalin (95%) were acquired from Alta Technology Co., Ltd. (Tianjin, China). 2-methylimidazole (98%) and PA (97%) were procured from McLean Biochemical Technology Co., Ltd. (Shanghai, China). TP (97%), PbCl_2_ (>99%) and Zn(NO_3_)_2_·6H_2_O (99%) were procured from Aladdin Biochemical Technology Co., Ltd. (Shanghai, China).

### 3.2. Synthesis of Composite

#### 3.2.1. Synthesis of ZIF-8

Synthesis of ZIF-8: Detailed information is provided in Text S1.

#### 3.2.2. Synthesis of ZIF-8@TPPa

ZIF-8@TPPa was synthesized using a solvothermal approach (Figure 1a). Initially, 13.4 mg of PA and 6 mg of ZIF-8 were dispersed in 25 mL of methanol through sonication. Subsequently, 17.4 mg of TP was introduced into the suspension, which was sonicated for 10 min to ensure homogeneous dispersion. Thereafter, 50 μL of saturated aqueous PbCl_2_ (10.8 g/L at 298 K), used as a catalyst, was added to the mixture, and the blend was vigorously shaken for 10 s. The resulting suspension was transferred to a stainless-steel autoclave, where it reacted at 120 °C for 12 h. Lastly, the obtained ZIF-8@TPPa was washed multiple times with anhydrous ethanol and desiccated at 60 °C for 24 h.

### 3.3. Characterization of Composite

Characterization of ZIF-8@TPPa: Detailed information is provided in Text S3.

### 3.4. Adsorption Experiments

The adsorption experiments were proceeded as follows: 20 mg of ZIF-8@TPPa was added to a centrifuge tube containing 20 mL of a mixed solution of three herbicides at a specific concentration, followed by shaking at 200 rpm for 30 min. After adsorption, the supernatant was harvested, passed through a 0.22 μm filter, and finally analyzed by liquid chromatography–tandem mass spectrometry (LC-MS/MS).

For adsorption kinetics tests, 20 mg of ZIF-8@TPPa was incorporated into 20 mL of herbicide solutions with a concentration of 200 mg/L. Triplicate samples were prepared for each condition. The mixtures were shaken for 2, 5, 10, 30, 60, 120, and 180 min, respectively. The adsorption data were evaluated using pseudo-first-order (PFO) (Equation (S1)) and pseudo-second-order (PSO) (Equation (S2)) kinetic models.

For the adsorption isotherm tests, 20 mg of the composite was added into 20 mL of herbicide solutions with diverse concentrations (10, 20, 30, 70, 120, 160, and 200 mg/L), with each concentration tested in triplicate. The suspensions were shaken at 200 rpm for 60 min. The equilibrium adsorption data were correlated with both the Langmuir (Equation (S3)) and Freundlich (Equation (S4)) model.

Thermodynamic adsorption tests were performed by introducing 20 mg of ZIF-8@TPPa into 20 mL of herbicide solutions with a concentration of 10 mg/L at temperatures of 15, 25, 35, and 45 °C. Each temperature condition was prepared in triplicate. The mixtures were shaken at 200 rpm for 60 min. Thermodynamic variables, including standard entropy change ΔS (kJ/(mol K)), standard enthalpy change ΔH (kJ/mol) and Gibbs free energy change ΔG (kJ/mol), were calculated in line with Equations (S5) and (S6).

## 4. Conclusions

In this research, a novel core–shell ZIF-8@TPPa was successfully synthesized for the efficient removal of herbicides from water. This composite exhibited adsorption capacities for butachlor (232.56 mg/g), anilofos (188.68 mg/g), and pendimethalin (285.71 mg/g), which were significantly higher than those of pristine TPPa or ZIF-8 alone. The adsorption process of ZIF-8@TPPa involved a synergistic mechanism combining monolayer chemisorption and multilayer physisorption. Notably, ZIF-8@TPPa overcame the acid stability limitation of pristine ZIF-8, maintaining high adsorption efficiency across a broad pH range (3–9) and under various ionic strengths. ZIF-8@TPPa also exhibited excellent renewability, with adsorption efficiency remaining above 80% after five cycles. These results demonstrate the great potential of ZIF-8@TPPa for herbicide removal in complex aquatic environments and provide new insights into the application of MOF@COF for environmental remediation.

## Figures and Tables

**Table 1:** Parameters of adsorption kinetics.

Herbicide	q_e,exp_ (mg/g)	Pseudo-First-Order Model			Pseudo-Second-Order Model		
		K_1_ (min^−1^)	q_e,cal_ (mg/g)	R^2^	K_2_ (g/(mg min))	q_e,cal_ (mg/g)	R^2^
butachlor	184.4 ± 2.15	0.0385	73.05 ± 1.36	0.8928	4.13 × 10^−4^	185.2 ± 1.82	0.9999
anilofos	173.2 ± 1.98	0.0391	18.84 ± 0.75	0.8578	2.60 × 10^−4^	172.4 ± 1.56	0.9999
pendimethalin	197.0 ± 2.32	0.0335	79.24 ± 1.41	0.7719	4.49 × 10^−4^	201.1 ± 2.05	0.9995

## Data Availability

Any further data may be obtained from the authors upon reasonable request.

## Supplementary Materials

The following supporting information can be downloaded at: https://www.mdpi.com/article/10.3390/molecules31111799/s1, Text S1: Synthesis of ZIF-8; Text S2: Synthesis of pure TPPa; Text S3: Characterization of ZIF-8@TPPa; Text S4: Detailed elution/regeneration procedure of ZIF-8@TPPa; Table S1: Molecular structure of herbicides; Table S2: BET data and removal rates of ZIF-8, TPPa, and ZIF-8@TPPa; Table S3: Model parameters for adsorption isotherms; Table S4: Parameters for adsorption thermodynamics; Figure S1: SEM morphology of ZIF-8@TPPa (A), and EDS elemental mapping images of all elements overlaid (B), N (C), C (D), O (E) and Zn (F). Figure S2: N_2_ adsorption isotherms and pore size distributions of ZIF-8@TPPa (a), ZIF-8 (b), and TPPa (c); Figure S3: Linear fitting results of adsorption thermodynamics (n = 3); Figure S4: Effects of various ionic interferences on the removal efficiency of butachlor, anilofos, and pendimethalin (data are presented as mean ± SD, n = 3); Figure S5: Removal efficiencies of pendimethalin (a), butachlor (b), and anilofos (c) by ZIF-8@TPPa in river water, seawater, and tap water (data are presented as mean ± SD, n = 3); Figure S6: Reusability of ZIF-8@TPPa over five cycles (data are presented as mean ± SD, n = 3); Figure S7: XRD patterns (a) of ZIF-8@TPPa before and after adsorption and SEM image (b) of ZIF-8@TPPa after adsorption. Reference [50].

## Abbreviations

The following abbreviations are used in this manuscript:

MOF	Metal–Organic Framework
COF	Covalent Organic Framework
SEM	Scanning Electron Microscopy
TEM	Transmission Electron Microscopy
XPS	X-ray Photoelectron Spectroscopy
XRD	X-ray Diffraction
FT-IR	Fourier-Transform Infrared Spectroscopy
PFO	Pseudo-First-Order
PSO	Pseudo-Second-Order
FWHM	Full-Width at Half-Maximum
