# Supplementary material for: Stable Core–Shell ZIF-8@TPPa Hybrids: Synthesis and Enhanced Herbicide Removal from Water
Source: Molecules. 2026 May 24;31(11):1799. doi: 10.3390/molecules31111799 (PMC13257848; doi:10.3390/molecules31111799)
Supplement: Supplementary file 1 [file molecules-31-01799-s001.zip › molecules-4278992-supplementary.pdf]

Supplementary Material

Stable Core–Shell ZIF-8@TPPa Hybrids: Synthesis and Enhanced Herbicide Removal from Water

Zeyuan Li [1,2,†], Zhenzhen Liu [2,†], Xiangping Lin [1], Mengyuan Ge [1], Nannan Wu [2], Xinquan Wang [2], Yuteng Zhou [2], Shuchun Wu [3], Wei Ding [1,*] and Peipei Qi [2,*]

[1] College of Plant Protection, Northeast Agricultural University, Harbin 150030, China; zeyuan1214@gmail.com (Z.L.); linxiangping2018@163.com (X.L.); gemengyuan0413@163.com (M.G.)

[2] State Key Laboratory for the Quality and Safety of Agro-Products & Institute of Agro-Product Safety and Nutrition, Zhejiang Academy of Agricultural Sciences, Hangzhou 310021, China;
18768479497@163.com (Z.L.); wunannan4089@163.com (N.W.); wangxq@zaas.ac.cn (X.W.); zhouyuteng1999@163.com (Y.Z.)

[3] Key Laboratory of Biomarkers and In Vitro Diagnosis Translation of Zhejiang Province, School of Laboratory Medicine and Bioengineering, Hangzhou Medical College, Hangzhou 310053, China; w_sc@163.com

* Correspondence: dingwei@neau.edu.cn (W.D.); qipeipei@zaas.ac.cn or qipeipei@zaas.cn (P.Q.);
Tel.: +86-451-551-90647 (W.D.); +86-571-864-19051 (P.Q.)

† These authors contributed equally to this work.

Text S1 Synthesis of ZIF-8

Firstly, 0.738 g of $Zn(NO_3)_2 \cdot 6H_2O$ was dissolved in 25 mL of methanol to prepare solution A, whereas 1.625 g of 2-methylimidazole was introduced into 25 mL of methanol to form solution B. Both solutions A and B were sonicated until homogeneous. Subsequently, solution A was added to solution B drop-by-drop under magnetic stirring. The resulting mixture was centrifuged at 12000 rpm for 10 minutes. The obtained ZIF-8 crystals were rinsed repeatedly with methanol and dried at 60 °C overnight.

Text S2 Synthesis of pure TPPa

TPPa was synthesized via a solvothermal method. Specifically, 13.4 mg of p-phenylenediamine and 17.4 mg of 2,4,6-trihydroxybenzene-1,3,5-tricarbaldehyde (TP) were dissolved in methanol, followed by ultrasonic treatment to form a homogeneous mixture. Subsequently, 50 μL of glacial acetic acid solution was added as a catalyst. The resulting suspension was transferred into a stainless steel autoclave and reacted at 120°C for 12 h. Finally, the as-obtained TPPa product was washed repeatedly with absolute ethanol and dried at 60°C for 24 h.

Text S3 Characterization of ZIF-8@TPPa

The structure and morphology of ZIF-8@TPPa were characterized using transmission electron microscopy (TEM, JEM-1400, Japan) and scanning electron microscopy (SEM, Gemin 300, Japan). Energy-dispersive X-ray spectroscopy (EDS) coupled with scanning electron microscopy (SEM) was employed to determine the elemental composition and to generate elemental distribution maps (C, N, O, and Zn) of ZIF-8@TPPa. The crystalline structure of ZIF-8@TPPa was characterized by powder X-ray diffraction (PXRD, Smartlab, Japan). The pore structure and specific surface area of ZIF-8@TPPa were characterized using a pore size and surface area analyzer (BET, Micromeritics 2460, USA). The elemental composition and chemical states were determined by XPS (XPS, K-Alpha, USA). The functional groups of the composite were analyzed using a Fourier Transform Infrared Spectrometer (FT-IR, Nicolet iS50, USA) in transmission mode via the KBr pellet method, where each pellet was prepared with a fixed total mass of 100 mg to ensure consistent thickness and uniformity. The spectra were collected over a range of 400-4000 cm^{-1} with a resolution of 4 cm^{-1} and 32 scans.

Text S4 The detailed elution/regeneration procedure of ZIF-8@TPPa

A measure of 20 mL of a mixed standard solution of herbicides with a concentration of 10 mg/L was aliquoted into a centrifuge tube. Subsequently, 20 mg of the ZIF-8@TPPa composite adsorbent was added. After dispersion, the mixture was placed in a constant-temperature shaker and shaken at 200 r/min for 30 min at room temperature. Following shaking, the supernatant was collected, passed through a 0.22 μm filter, and finally analyzed by liquid chromatography–tandem mass spectrometry (LC-MS/MS). The remaining aqueous solution was discarded, and 20 mL of ethanol was added to elute the adsorbent, followed by ultrasonic treatment for 10 min. The mixture was centrifuged to remove the ethanol eluate, and this elution process was repeated three times. The adsorbent was then placed in a vacuum oven and dried at 60 °C for 6 h. Subsequently, the adsorption experiment was repeated five times following the aforementioned procedure.

$$\ln\left(Q_e - Q_t\right) = \ln Q_e - k_1 t \quad \textbf{(Eq. S1)}$$

$$\frac{t}{Q_t} = \frac{1}{k_2 Q_e^2} + \frac{t}{Q_e} \quad \textbf{(Eq. S2)}$$

Here, K_1 (min^{-1}) is a constant of the pseudo-first-order adsorption rate, K_2 (g mg^{-1} min^{-1}) is a constant of the pseudo-second-order rate, and

q_t (mg/g) is the amount of analyte adsorbed on the absorbent at time t (min).

$$\frac{C_e}{q_e}=\frac{1+bC_e}{bq_{max}} \quad \textbf{(Eq. S3)}$$

$$\ln q_e=\ln K_f+\frac{1}{n}\ln C_e \quad \textbf{(Eq. S4)}$$

Here, q_{max} (mg/g) and b (L/mg) denote the maximum adsorption capacity and adsorption rate, respectively, q_e (mg/g) refers to the amount of herbicide adsorbed by the adsorbent at equilibrium, K_f ((mg/g) (L mg^{-1})$^{1/n}$) is a constant reflecting the relative adsorption capacity, and n is a dimensionless constant reflecting the adsorption intensity and surface heterogeneity.

$$\Delta G=-RTLnK \quad \textbf{(Eq. S5)}$$

$$LnK=-\frac{\Delta H}{RT}+\frac{\Delta S}{R} \quad \textbf{(Eq. S6)}$$

Here, K_0 is the thermodynamic equilibrium constant, derived from the ratio of the equilibrium adsorption capacity (q_e, mg/g) to the equilibrium concentration (C_e, mg L^{-1}); R represents the universal gas constant (8.314 J/(mol K)); and T(K) denotes the absolute temperature in Kelvin.

Table S1. Molecular structure of herbicides.

Herbicides	Structural formula	Molecular weight (g/mol)
butachlor	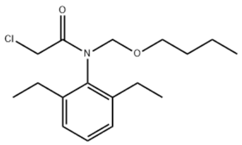	311.8
anilofos	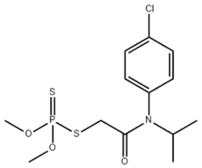	367.9
pendimethalin	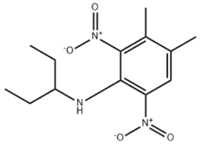	281.3

Table S2. BET date and removal rate of ZIF-8, TPPa, and ZIF-8@TPPa.

Materials	BET data			Removal rate (%)		
	BET specific surface (m^2/g)	Total pore volume (cm^3/g)	Pore diameter (nm)	Pendimethalin	Anilofos	Butachlor
ZIF-8	1700.8	0.141	2.53	/	90±0.9	85±0.8
TPPa	137.1	0.499	5.21	74±0.3	/	80±0.5
ZIF-8@TPPa	162.2	0.164	5.19	99±0.1	93±2.4	90±1.3

Data are mean ± SD, n = 3.

Table S3. Model parameters of adsorption isotherm.

Herbicide	Langmuir model				Freundlich model			
	b (L/mg)	q_{max} (mg/g)	R^2	fitting equation	k_f (mg/g)(L/mg)$^{1/n}$	1/n	R^2	fitting equation
butachlor	0.184±0.012	232.56±5.21	0.9863	y=0.0043x+0.0234	33.54±2.15	0.6536±0.021	0.9866	y=0.6536x+3.5127
anilofos	0.127±0.010	188.68±4.33	0.9767	y=0.0051x+0.0471	23.54±1.88	0.5714±0.019	0.9825	y=0.5714x+3.1587
pendimethalin	0.700±0.025	285.71±6.45	0.9785	y=0.0035x+0.0050	104.67±5.62	0.7143±0.024	0.9905	y=0.7143x+4.6508

Table S4. Parameters of adsorption thermodynamics.

pesticides	*ΔG (kJ/mol)*				*ΔH (kJ/mol)*	*ΔS (kJ/mol K)*
	**288.15K**	**298.15K**	**308.15K**	**318.15K**		
butachlor	-5.03	-7.17	-9.30	-11.4	56.41	213.24
anilofos	-5.19	-6.98	-8.77	-10.6	46.34	178.84
pendimethalin	-9.61	-10.7	-11.8	-12.8	21.25	107.10

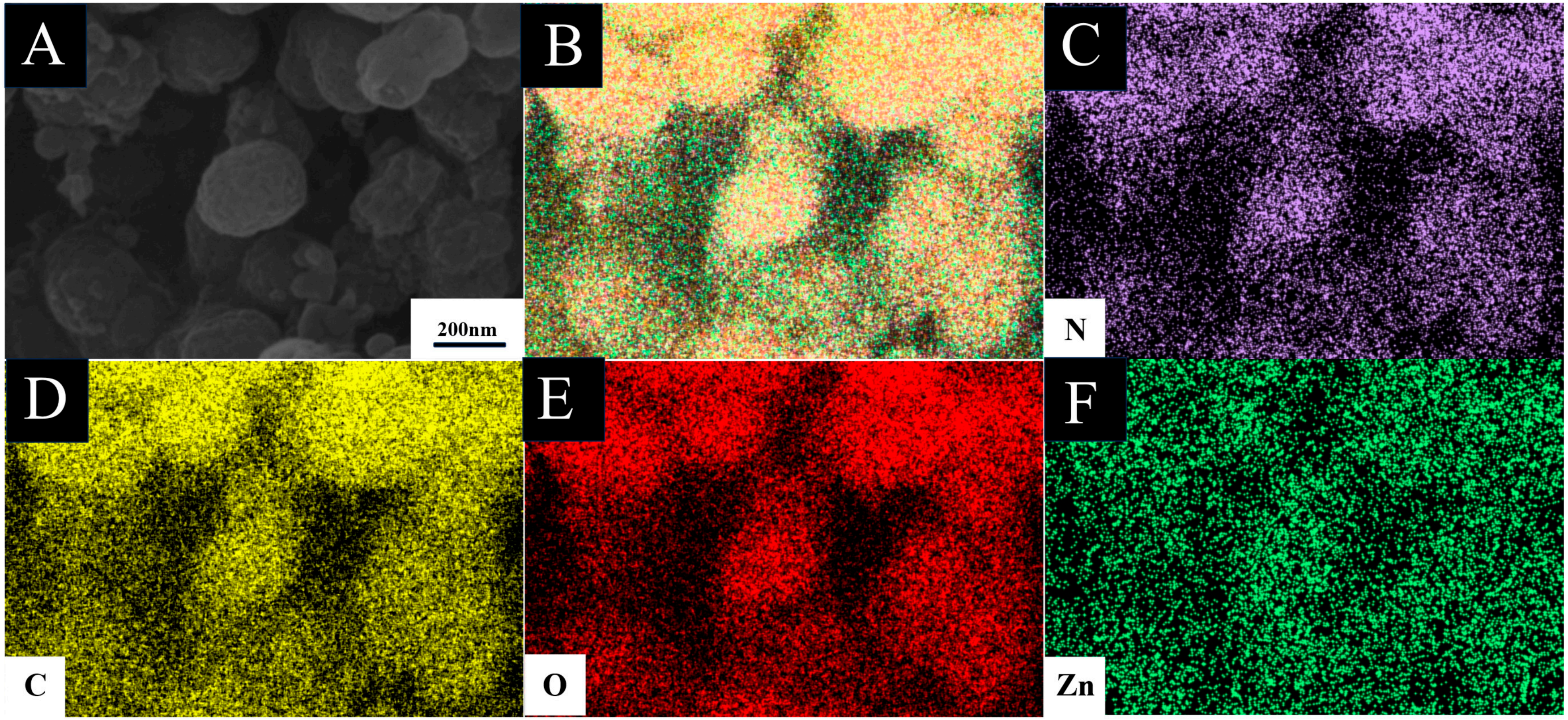

Figure S1. SEM morphology of ZIF-8@TPPa (A), and EDS elemental mapping images of all elements overlay (B), N (C), C (D), O (E), and Zn (F).

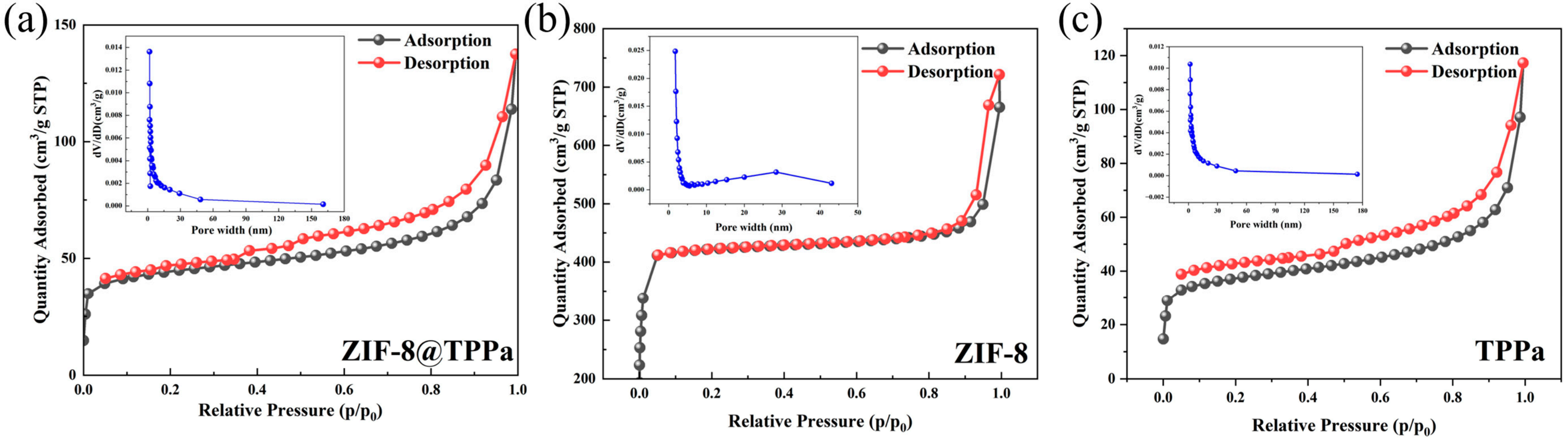

Figure S2. N_2 adsorption isotherms and pore size distributions of ZIF-8@TPPa (a), ZIF-8 (b), and TPPa (c).

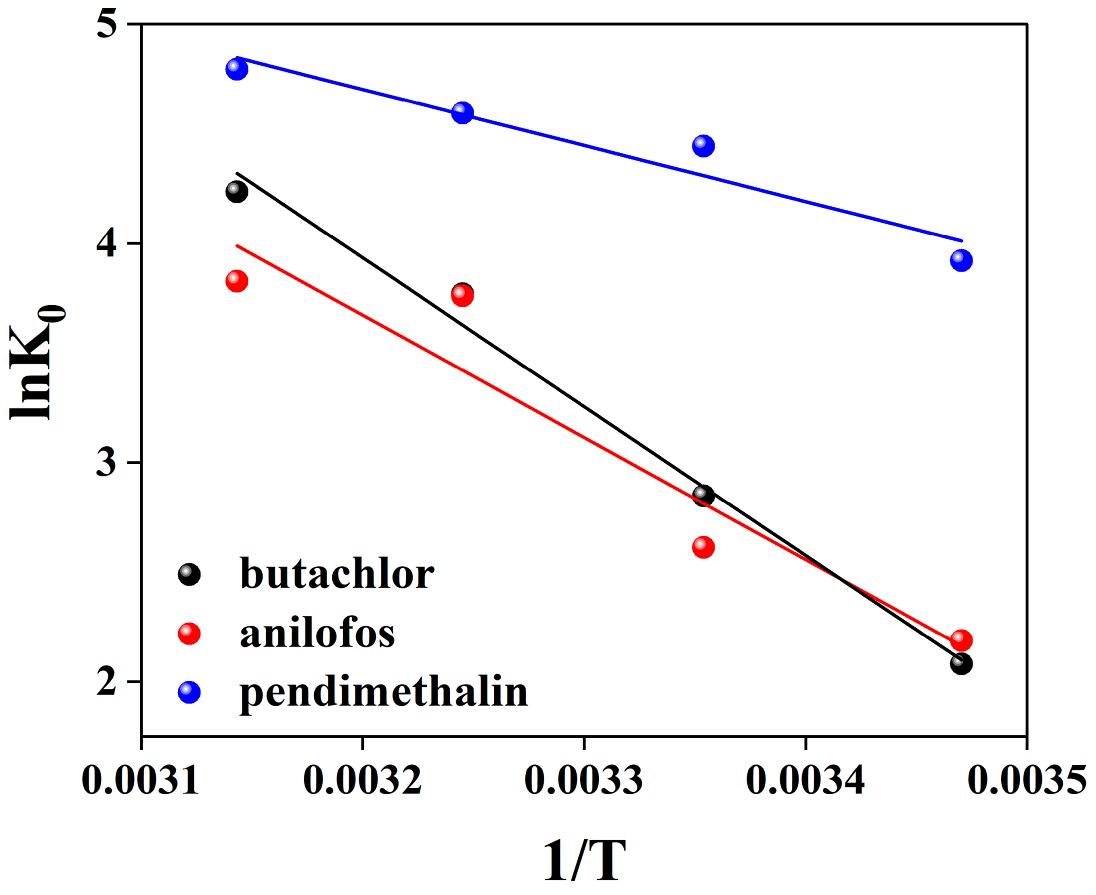

Figure S3. Linear fitting results of adsorption thermodynamics (n=3).

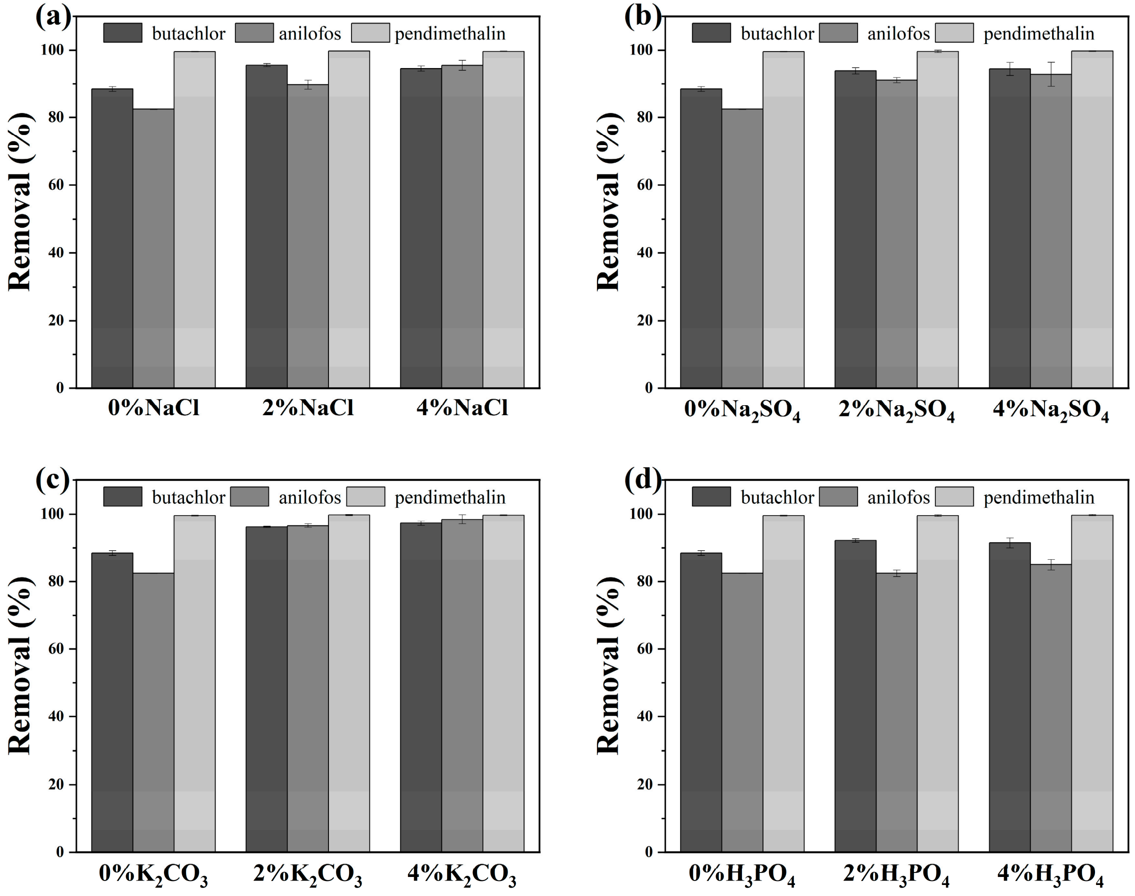

Figure S4. Effect of various ionic interference on the removal efficiency of butachlor, anilofos, and pendimethalin (data are mean ± SD, n = 3).

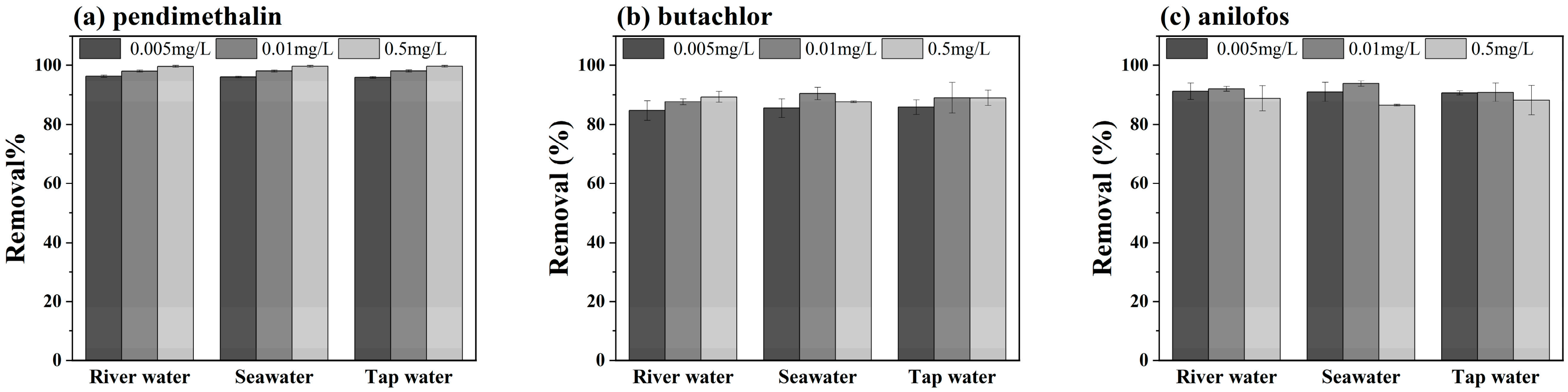

Figure S5. Removal efficiency of pendimethalin (a), butachlor (b), and anilofos (c) by ZIF-8@TPPa in river water, seawater, and tap water (data are mean ± SD, n = 3).

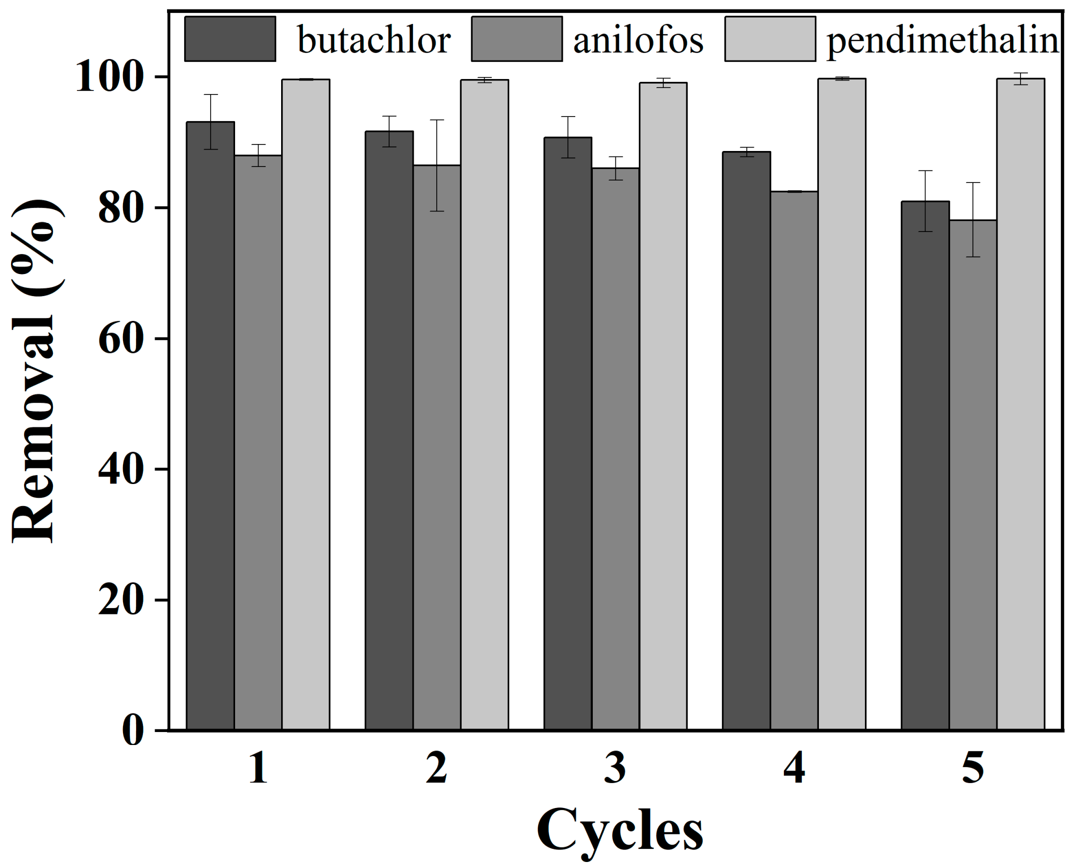

Figure S6. The reusability of ZIF-8@TPPa, demonstrated for five cycles (data are mean ± SD, n =3).

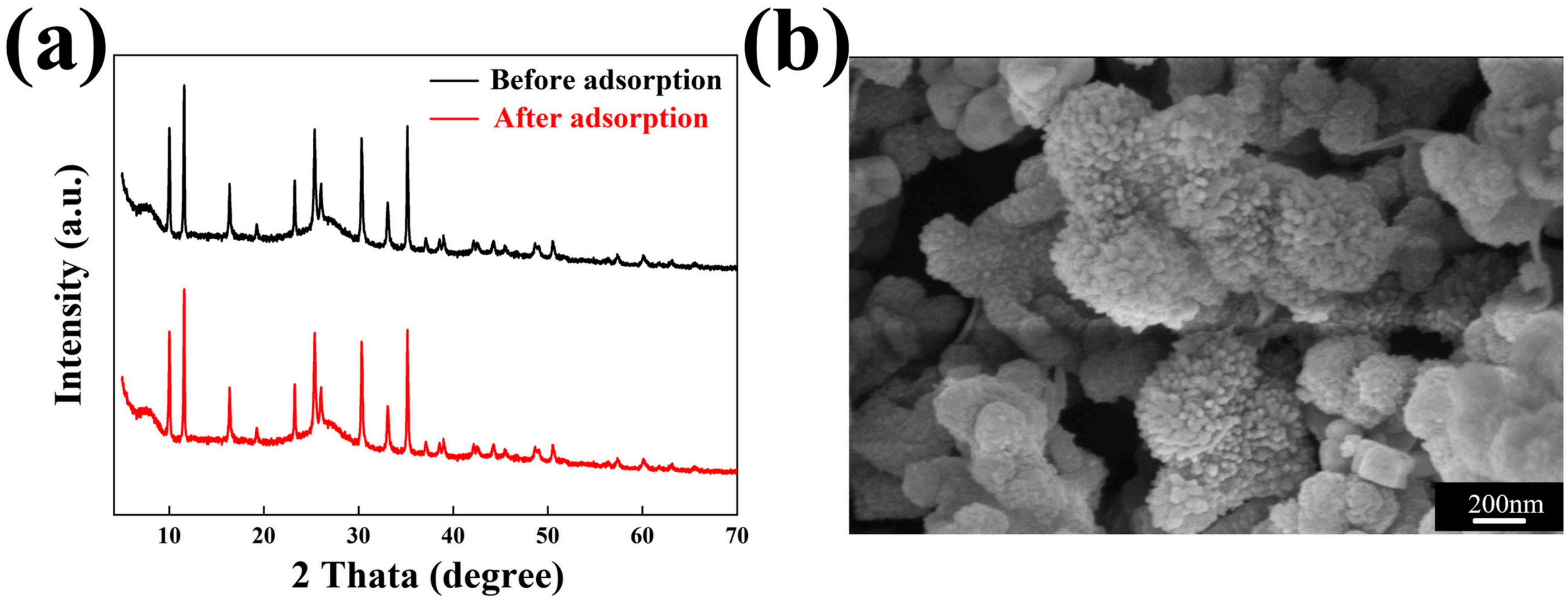

Figure S7. XRD patterns (a) of ZIF-8@TPPa before and after adsorption; SEM image (b) of ZIF-8@TPPa after adsorption.

References

S. Karak, S. Kandambeth, B.P. Biswal, H.S. Sasmal, S. Kumar, P. Pachfule, R. Banerjee, Constructing Ultraporous Covalent Organic Frameworks in Seconds via an Organic Terracotta Process, J. Am. Chem. Soc., 139(2017) 1856-1862.
